# Supplementary material for: Eliciting preferences for outpatient care experiences in Hungary: A discrete choice experiment with a national representative sample
Source: PLoS One. 2020 Jul 31;15(7):e0235165. doi: 10.1371/journal.pone.0235165 (PMC7394384; doi:10.1371/journal.pone.0235165)
Supplement: S1 File — (PDF) [file pone.0235165.s002.pdf]

S1 File. DCE survey in Hungarian and translation to English.

Hungarian version

Az emberek elvárásai az orvosi ellátással kapcsolatban különbözőek lehetnek attól függően, hogy ki mit tart fontosnak.
A következőkben szeretnénk megismerni az Ön véleményét egy sor olyan helyzetről, amelyekbe az emberek az orvosi ellátás során kerülhetnek.
Az Ön véleményére vagyunk kíváncsiak. Nincsenek jó vagy rossz válaszok.
Képzelje el, hogy Önnek az egészségével kapcsolatos problémája van, ami aggasztja Önt, de nem igényel azonnali ellátást. Konzultáció vagy kivizsgálás céljából szakorvoshoz szeretne fordulni ezzel a problémával.
A következőkben 5 választási helyzetet mutatunk be Önnek, mindegyikben kétféle orvosi ellátás szerepel. Azt szeretnénk megtudni, hogy Ön melyik orvosi ellátást szeretné inkább.
Az orvosi ellátásokat néhány jellemzővel írjuk le. Egyik jellemző például, hogy az orvos teljes mértékben / közepesen / egyáltalán nem elegendő időt tölt Önnel a vizsgálat során. Az ellátások abban különböznek egymástól, hogy milyen szinten érintettek az egyes jellemzők az adott leírásban.
Arra kérjük Önt, válassza ki a két orvosi ellátás közül az Önnek jobban tetszőt.

Melyik orvosi ellátást szeretné inkább? Kérjük, kattintson rá a kiválasztott orvosi ellátásra!

Orvosi ellátás A	Orvosi ellátás B
Ön 12 hét (3 hónap) múlvára kap időpontot az orvosi vizsgálatra.	Ön másnapra kap időpontot az orvosi vizsgálatra.
Az orvosi ellátás napján 2 órát kell várakoznia, mielőtt sorra kerül.	Az orvosi ellátás napján 4 órát kell várakoznia, mielőtt sorra kerül.
Az orvos nem tölt elegendő időt Önnel a vizsgálat során.	Az orvos elegendő időt tölt Önnel a vizsgálat során.
Az orvos úgy magyarázza el a dolgokat, hogy Önnek azt könnyű megérteni.	Az orvos úgy magyarázza el a dolgokat, hogy Önnek azt nem könnyű megérteni.
A orvos nem ad lehetőséget Önnek, hogy feltegyen kérdéseket vagy felvesse az aggodalmait a javasolt kezeléssel kapcsolatban.	A orvos lehetőséget ad Önnek, hogy feltegyen kérdéseket vagy felvesse az aggodalmait a javasolt kezeléssel kapcsolatban.
Az orvos bevonja Önt az ellátásával és kezelésével kapcsolatos döntésekbe olyan mértékben, amennyire Ön akarja.	Az orvos nem vonja be Önt az ellátásával és kezelésével kapcsolatos döntésekbe olyan mértékben, amennyire Ön akarja.
A vizsgálat Önnek 5 000 Ft-ba kerül.	A vizsgálat Önnek 0 Ft-ba kerül.
○	○

[Four more choice tasks followed. The blocks that were randomly assigned to the participants are made available as S2 File]

Milyen mértékben ért egyet az alábbi állítással?
Nehéz volt megválaszolni az Orvosi ellátás 'A' és Orvosi ellátás 'B' közötti választásokkal kapcsolatos kérdéseket.

				Egyet is értek meg nem is				
Egyáltalán nem értek egyet (Nem volt nehéz)	⊙1	⊙2	⊙3	⊙4	⊙5	⊙6	⊙7	Teljes mértében egyetértek (nehéz volt)

Miért volt nehéz megválaszolni a kérdéseket? (Több választ is megjelölhet)

☑ 1. Nehéz volt megérteni a különböző orvosi ellátással kapcsolatos helyzeteket.
☑ 2. Nehéz volt elképzelni, hogy orvosi ellátásra van szükségem.
☑ 3. Nehéz volt választani a két orvosi ellátás között.
☑ 4. Nehéz volt elolvasni a különböző orvosi ellátásokat jellemző leírásokat.
☑ 5. Egyéb okból

Translation to English

People's expectations for medical care may vary, depending on what they find important. In the next section, we would like to know your opinion about a series of situations people may face during medical care.
We are interested in your opinion. There are no good or bad answers.
Imagine that you have a health problem that concern you but does not require immediate care and to receive health care you will be visiting a specialist for a consultation or an examination.
We will present five pairs of options for you to choose from, each with two types of medical care. We want to know which medical care you would prefer.
Each medical care is described with some characteristics. For example, one of the characteristics is that your doctor spends/does not spend enough time with you during the visit. Medical care options differ in the degree to which each characteristic is affected in the description.
We ask you to choose which of the two medical care scenarios you prefer.

Which medical care would you prefer? Please click on the selected medical care!

Medical care "A"
You have a medical appointment in **12 weeks (3 months)**.
On the actual day of the consultation, you have to **wait 2 hours** before you are actually seen.
The doctor **does not spend enough time** with you during the consultation.
The doctor explains things in a way that is **easy for you to understand.**
The doctor **does not give you an opportunity** to ask questions or raise concerns about recommended treatment.
The doctor **involves you as much as you wanted** to be in decisions about your care and treatment.
The consultation costs you **5000 HUF**.
○

Medical care "B"
You have a medical appointment the **next day.**
On the actual day of the consultation, you have to **wait 4 hours** before you are actually seen.
The doctor **spends enough time** with you during the consultation.
The doctor explains things in a way that **is not easy to understand**.
The doctor **gives you an opportunity** to ask questions or raise concerns about recommended treatment.
The doctor **does not involve you as much as you wanted** to be in decisions about your care and treatment.
The consultation costs you **0 HUF**.
○

[Four more choice tasks followed. The blocks that were randomly assigned to the participants are made available as S2 File]

To what extent do you agree with the following statement?

It was difficult to answer the questions about the choices between Medical Care 'A' and Medical Care 'B'.

				Nor agree or disagree				
I do not agree at all (It was not difficult)	⦿1	⦿2	⦿3	⦿4	⦿5	⦿6	⦿7	I totally agree (it was difficult)

Why was it difficult to answer the questions? (You can mark multiple answers)

- ☑ 1. It was difficult to understand the different medical scenarios.
- ☑ 2. It was difficult to imagine the need for medical care.
- ☑ 3. It was difficult to choose between the two scenarios.
- ☑ 4. It was difficult to interpret the description of medical treatment in the two scenarios.
- ☑ 5. Other reasons.
