# Supplementary material for: Eliciting preferences for outpatient care experiences in Hungary: A discrete choice experiment with a national representative sample
Source: PLoS One. 2020 Jul 31;15(7):e0235165. doi: 10.1371/journal.pone.0235165 (PMC7394384; doi:10.1371/journal.pone.0235165)
Supplement: Attachment — Submitted filename: Response to Reviewers.docx [file pone.0235165.s004.docx]

**Response to Reviewers**

**Comments from the Editor**

**E1. Please ensure that your manuscript meets PLOS ONE's style requirements, including those for file naming.**

We proceeded accordingly.

**E2. Please include additional information regarding the survey or questionnaire used in the study and ensure that you have provided sufficient details that others could replicate the analyses. For instance, if you developed a questionnaire as part of this study and it is not under a copyright more restrictive than CC-BY, please include a copy, in both the original language and English, as Supporting Information.**

We provide as supporting information the DCE survey in its original language (Hungarian) and a translation to English (S1_File).

**E3. Please provide your institutional email address.**

We proceeded accordingly.

**E4. We note that you have indicated that data from this study are available upon request. PLOS only allows data to be available upon request if there are legal or ethical restrictions on sharing data publicly. For information on unacceptable data access restrictions, please see** [**http://journals.plos.org/plosone/s/data-availability#loc-unacceptable-data-access-restrictions**](https://linkprotect.cudasvc.com/url?a=http%3a%2f%2fjournals.plos.org%2fplosone%2fs%2fdata-availability%23loc-unacceptable-data-access-restrictions&c=E,1,T0NgyTROfWHvCiFWbnCgEEfY2OAdD-ra8DbxrjUqpfqqzrK1IoLb0DTcPVCLTUZ9nwBxepd9U5OEgZVKCiJACkca2aG5EyMCqAZDiawffAnAmCxuKw,,&typo=1)**.
In your revised cover letter, please address the following prompts:
a) If there are ethical or legal restrictions on sharing a de-identified data set, please explain them in detail (e.g., data contain potentially identifying or sensitive patient information) and who has imposed them (e.g., an ethics committee). Please also provide contact information for a data access committee, ethics committee, or other institutional body to which data requests may be sent.
b) If there are no restrictions, please upload the minimal anonymized data set necessary to replicate your study findings as either Supporting Information files or to a stable, public repository and provide us with the relevant URLs, DOIs, or accession numbers. Please see** [**http://www.bmj.com/content/340/bmj.c181.long**](https://linkprotect.cudasvc.com/url?a=http%3a%2f%2fwww.bmj.com%2fcontent%2f340%2fbmj.c181.long&c=E,1,4PKEFTo5gm6yrXeDxMXKRUQtRQsvIB0lc5EqVnLtppVw7fn7aUL02IHMfZfhjxVlKV3ghXCKV08sIY20wyvAz72H3wQzuMwzxLTHtA6RHpAcslQfIEfofGszV9XF&typo=1) **for guidelines on how to de-identify and prepare clinical data for publication. For a list of acceptable repositories, please see** [**http://journals.plos.org/plosone/s/data-availability#loc-recommended-repositories**](https://linkprotect.cudasvc.com/url?a=http%3a%2f%2fjournals.plos.org%2fplosone%2fs%2fdata-availability%23loc-recommended-repositories&c=E,1,aY23__ZIACDpW9MDBGgl-zgm3HEXlDMzreLD2glf7LZk2Agatc4YG80RL-U5h-2OVnHNFF-8w6Yyhn0GSx2EgbnTpbqjm80tKih5sAB6zA,,&typo=1)**.
We will update your Data Availability statement on your behalf to reflect the information you provide.**

We provide as supporting information a minimal anonymized dataset (S1_Dataset).

**Comments from Reviewer 1**

**R1C1. For International readers it would be helpful to have a brief background to primary health care in Hungary. For example the order of magnitude for co-payments or out of pocket costs would help the reader put the WTP values into context. The WTPs for aspects of care for non threatening health issue seem very high.**

We introduced a paragraph in the Introduction section to address this comment. It reads as follows:

“The Hungarian health system is organized around a single health insurance fund, which provides health coverage for nearly all residents. However, the benefit package is less comprehensive than in most European Union (EU) countries, and thus, a large number of people rely on out-of-pocket payments to access care [6]. Public health expenditure accounts for two-thirds of the total health expenditure, which sets the levels of out-of-pocket payments to almost double of the EU average (27% vs 16%) [6]. Out-of-pocket payments have been increasing partly because of the rising co-payments with pharmaceuticals and outpatient care, growing utilization of care providers in the private sector and the prevalence of informal payments [7]. Given this context, citizens’ experiences of care may be undermined up to some extent.” (Line 82–91)

We believe that with the given additional information an international reader may have a better understanding of the Hungarian context, especially regarding the relevance of increasing out-of-pocket payments in the Hungarian health care system. In addition, the references used in this paragraph may be of use for a reader who wishes more in-depth information about the Hungarian health system.

**R1C2. Some attribute levels seem very high and fall way beyond a realistic scenario. For example 3 month wait for appointments, 94 euros cost, 4 hour wait time. Using such extremes could lead to biased estimates of attribute preference and hence WTP. Is timely access to primary health care an issue in Hungary?**

We understand the concern of the Reviewer in regard to the breadth of some attribute levels, as they may seem unreasonable with the lenses of other contexts. However, they fit quite well to the Hungarian context. The health expenditure in Hungary is significantly below the EU average and only two-thirds are publicly funded. As such, Hungary has one of the highest levels of out-of-pocket payment in the EU and it represents almost twice as that of the EU average. In addition, the health benefit package is limited, which contributes to increasing out-of-pocket costs. Moreover, shortages and uneven distribution of health care professionals across the country undermine access to health services, especially outpatient services, which has been contributing for a growth in the utilization of private care services.

Given that there are very weak protection mechanisms in place to deal with the problem of increasing out-of-pocket payments, this is a great concern especially to most vulnerable populations. These were also the findings of a recent study that used the same data as we did in our DCE manuscript, where 27% of the respondents reported forgone medical visit due to difficulties in travelling, 24% unfilled prescriptions due to cost, 21% forgone medical appointments due to cost and 17 % skipped medical examinations due to costs (DOI: [10.1007/s10198-019-01063-0](https://www.researchgate.net/deref/http%3A%2F%2Fdx.doi.org%2F10.1007%2Fs10198-019-01063-0?_sg%5B0%5D=bA2hDtKpZcQpbvj48U2Lms46yJtx5eFP9h57ByVny5BLSwV1ijqIX9DsxkYBtfeLs1GkspTd5pHreuDWfHqZCX4hug.zv2EncFsbdi_HgzcXFbJpx9NiJ06mmNPxlzICrmsCJ9_S1k1_sraI04XooIfV_FeDLG7SVG3NS8nnDdH7wqwtQ)). A reporting on unmet medical needs because of waiting times is being prepared; preliminary findings were reported elsewhere (DOI: [10.1016/j.jval.2019.09.2097](https://www.researchgate.net/deref/http%3A%2F%2Fdx.doi.org%2F10.1016%2Fj.jval.2019.09.2097?_sg%5B0%5D=ccjEcU6Bj63y_NcF_6QUTxJS0LBWA2rf31ADv0yV16ETllBZoOdd1_oC_r_YX4nDQlbrtQxW0EdBhxbDAEH_bU0mIw.n9oqYTAs8h_bY-MhxYYGmeZTKmNyia1udNwuQp-cXykxA5gL8iEgd5XoYzaen_FvrzXFO4n845x6D15ri2-WNQ)) and suggest that waiting time for an appointment or at a doctor’s office is common and frequently a problem to citizens.

**R1C3. It is not clear why the authors have chosen both conditional and mixed logit models. This should be justified in the methods section. The more complex mixed logit does not provide any additional information to support the findings and does not seem to be justified.**

Following the suggestion of the Reviewer, we have provided further information in the text to justify the use of both conditional and mixed logit models. The first two models (conditional logit and mixed logit with price attribute to be fixed) were included as benchmark model specifications and with the purpose of providing insights in regard to sensitivity testing using varying model specifications. Changes in text read as follows:

“Both model 1 and 2 were included as benchmark model specifications, where the latter is still quite common in the DCE literature because it allows the computation of willingness to pay estimates in preference space in a straightforward manner [20]. To improve the realism of model assumptions, in model 3 we have also specified the out-of-pocket coefficient to be log-normally distributed allowing the preference for this attribute to vary across respondents.” (Line 349–354)

**R1C4. The method used for sub group analyses should be explained. I assume models were run on separate data sets? If so as noted in the limitation this is not an ideal approach as individual respondents will be present in multiple sub groups. E.g. age and gender, education and income. Given the large data set, the authors could have considered inclusion of interaction variable for major sub groups on a trial and error basis. Or again given the large sample size a latent class model would have been a better approach to addressing preference heterogeneity than sub group analysis. I would recommend that the authors consider alternate approaches.**

After thoughtful consideration, we have decided to not report on these analyses. The Reviewer was correct to assume that the sub-group analyses were run on separate datasets. We now share similar concerns with this parsimonious segmentation approach, which may be very misleading. As suggested, we ran different model specifications with the inclusion of interaction variables in preference and WTP space. However, we faced several challenges in regard to computational power and the convergence of the models. Notwithstanding, we took note of the suggestion of using a latent class model, which we will consider in future studies.

**R1C5. The probability calculations and plot are confusing. The base case that has a 4% probability is clear, however what attribute levels are used to produce the curve is not clear. It maybe that I am not following the method. Irrespective in my view the probability calculations do not add to the findings. Rather the WTP provide a simpler estimate of attribute importance.**

After thoughtful consideration, we have decided to remove this component of the manuscript and re-focus the manuscript to WTP measures. Thank you.

**R1C6. Table 1 should include comparison to general population distributions for Hungary as this was a stated aim of recruitment.**

We added a column to Table 1 where we show the distribution of the general adult population of Hungary across the socio-demographic variables considered, for which we considered data from the micro-census held in 2016. All data were retrieved from the Hungarian Central Statistical Office. We removed the lines with information on the distribution of the sample by income tertiles from the Table, as these data will not be of use for the following sections. However, we provide a reference in the text where readers can access further information about the sample.

**Comments from Reviewer 2**

**R2C1. The methodology section would benefit from reorganisation to ensure a proper flow that readers can follow. The authors can learn from guidelines on how to report choice experiments such as Bridges et al. 2011** [**https://doi.org/10.1016/j.jval.2010.11.013**](https://linkprotect.cudasvc.com/url?a=https%3a%2f%2fdoi.org%2f10.1016%2fj.jval.2010.11.013&c=E,1,gaMpuzl3JaRnNv9oKHUq7Nvc762TYWPn5mW3yZVwnQW1H7V7NKhkAxCyTeJTQcZ4_53ToWJSm9uPNmfWp9bYqX_OJiH_UdD7GQlvpWwCSShS1nutvbWg&typo=1)

We have made several changes throughout the manuscript, especially in the Methods section, to enhance the flow and reading experience of the manuscript.

**R2C2. Deriving attributes and levels is a very important step of a choice experiment. The authors need to comprehensively explain in the text how they arrived to the selected attributes and levels. Were literature reviews and qualitative studies conducted? How did they reduce the number of attributes and levels (were experts engaged, were patients involved?). Explain these aspects in the text**

We added further in text information about attribute selection. It reads as follows:

**“**The attribute selection for aspects of the care experience that add value to patients was based on the OECD’s proposed set of questions to gauge PREMs in outpatient settings [5]. Following best practices of attribute identification and selection [14], we chose those PREMs because of several reasons: 1) a recent systematic review often identified those measures in DCE studies to elicit patients’ preferences for primary health care [15]; 2) previous research has identified strong linkages between those attributes and quality of care, clinical safety and effectiveness [16, 17]; 3) those attributes are strong predictors of one’s perception of quality of an outpatient consultation [18], which may be an important consideration when choosing a consultation and; 4) those attributes represent a balance between what is relevant for patients and the health policy context [15]. Attributes covered aspects such as those of people’s access to care (e.g. waiting time for an appointment and waiting time at a doctor’s office) and experiences with outpatient care. About the latter, the attributes focused on aspects of care such as those of a doctor spending enough time with a patient, providing easy to understand information, giving a patient opportunity to ask questions or raise concerns about recommended treatments, and involving a patient in decision making about care and treatment. Additionally, we used a price attribute (out-of-pocket payments) to compose each outpatient consultation scenario (Table 1).” (Line 175–192)

In addition, we highlight that the PREMs statements were validated to the Hungarian language (process reported in a previous study DOI: 10.1007/s10198-019-01064-z) and that in the paper-based pilot of the DCE study the attributes and attribute levels were discussed with the participants.

**R2C3. The study lacks an opt-out or status quo Can the authors provide a very good justification in the text for excluding either one of these? This is because the lack of an opt-out or status quo exposes your WTP estimates to criticism.**

We have provided more detailed information in text on the reasoning for not including an opt-out or status quo option. It reads as follows:

“The DCE module started with a brief explanation about what was expected from the respondents regarding the choice tasks (S1 File). Afterward, respondents were instructed the following: “Imagine that you have a health problem that concern you but does not require immediate care and to receive health care you will be visiting a specialist for a consultation or an examination.” Next, respondents were asked to choose between two different outpatient consultation scenarios (*A* or *B*). All the tasks that were presented to the respondents for preference elicitation included all attributes, i.e. each consultation scenario was presented as full profile. We did not incorporate an opt-out or a status quo option. The inclusion of an opt-out option was not adequate given that in the task instructions provided to respondents we assumed that they would seek care because of a concerning health problem. Although an opt-out option might have reduced bias in parameter estimates, it would jeopardize a better understanding on respondents’ preferences if a large number of respondents choose the opt-out option. In addition, a *status quo* option was not included because this study aimed to estimate trade-offs between characteristics of a medical consultation (e.g. a doctor spending enough time in consultation with a patient or providing easy to understand explanations) rather than the expected uptake of certain consultations.” (Line 209–225)

**R2C4. What is the justification of the choosing the D-efficient design over other designs that exist such as Bayesian efficient designs? Which software was used to generate the experimental design? Researchers should explain these issues in the text.**

We are aware of the use of alternative approaches such as that of Bayesian efficient designs. However, that approach is not yet widespread as that of D-efficiency, as suggested elsewhere (DOI: 10.1007/s40273-018-0734-2), where the proportion of DCE studies using Bayesian D-efficiency was of 8% (n=23) in contrast with 35% (n=105) of those that followed a D-efficiency design. Hence, we have decided for an approach that is more familiar to readers of this type of studies and less technically demanding for one of our target audiences: policy-makers.

Following the comment of the Reviewer, we included the following in text:

“For the study to become feasible we defined a D-efficient fractional design with priors set to zero, for main effects only, with adequate level balance and minimum overlap of attribute levels. We used Stata’s *dcreate* command to maximize the D-efficiency of the design based on the covariance matrix of conditional logit model.” (Line 227–231)

**R2C5. How did the authors derive their prior parameters for the D-efficient design? Did they use educated best guess or were the priors derived from the pilot study they conducted? The authors need to explain this in the text. Furthermore, which model did the authors optimise for in the experimental design? MNL, MMNL etc**

We have provided more detailed information on the approach used. It reads as follows:

“For the study to become feasible we defined a D-efficient fractional design with priors set to zero, for main effects only, with adequate level balance and minimum overlap of attribute levels. We used Stata’s *dcreate* command to maximize the D-efficiency of the design based on the covariance matrix of conditional logit model.” (Line 227–231).

**R2C6. Line 73: Though the sample was obtained from a panel of an internet survey company, the authors need to be a bit detailed on how the quota sampling approach had been implemented to sample the respondents. Explain this in the text.**

We provided further information about the sampling approach in the text, as suggested. It reads as follows:

“The recruitment process aimed at a target sample size of 1000 respondents. A disproportionate stratified random sampling was employed to reflect the characteristics of the general adult population of Hungary in terms of sex, age (by age group: 18–24, 25–34, 35–44, 45–54, 55–64 or 65 and over years), highest education level attained (primary, secondary or tertiary), type of settlement (Budapest, other cities or village) and region of residence (Central, Eastern or Western Hungary). Given that this was an online survey and that the use of the internet is lower among people aged 65 and older, the sampling aimed to reflect a fair representativeness of older age groups in comparison with the distribution of older age strata in the general adult Hungarian population. We used publicly available information of the Hungarian Central Statistical Office to characterize the distribution of the general adult population [11].” (Line 120–131)

**R2C7. Line 172 “we assumed error terms to be independently and identically distributed following a logistic regression". I suggest the authors should replace the term "logistic regression" with "type 1 extreme value distribution".**

We agree with the suggestion of the reviewer and changed the text as suggested. It now reads as follows:

“We assumed errors to be independent and identically distributed following a type-one extreme value distribution.” (Line 337–338)

**R2C8. Line 181 assuming the parameter of the out-of-pocket payment attribute as fixed rather than random is misleading. It suggests that the standard deviation of unobserved utility of the out-of-pocket attribute is the same for all observations. The authors should rerun their mixed multinomial logit model with the out-of-pocket payment attribute assuming a random and lognormal distribution instead of fixed.**

We understand that assuming preference homogeneity for the out-of-pocket payment may be misleading and often unrealistic. However, considering a price attribute to be fixed is still a common approach to dealing with the challenges of computing WTP out of the ratio of two randomly distributed terms (DOI: 10.1007/s00181-011-0500-1). For this reason, we have decided to preserve the reporting of such model (model 2). Notwithstanding, we agree with the Reviewer that to account for heterogeneity in preferences we should have ran the analyses considering the out-of-pocket coefficient random and log-normally distributed. We accounted for this suggestion with model 3, as reported in the manuscript.

**R2C9. Line 197-line 205. The authors should make it clear here that they computed the WTP measures in preference space using the conditional logit model coefficients. However, the authors still compute WTP estimates in preference space using the Mixed Multinomial Logit Model coefficients. They assume that the out-of-pocket payment attribute parameter is fixed and compute WTP as a ratio of parameters (-attribute/out-of-pocket) which is known as preference space. However, This can result in WTP distributions that are not well behaved as the authors are not accounting for variability in the price attribute (our-of-pocket payments). Therefore, the authors have to rerun the WTP estimates for mixed multinomial logit model in WTP space with the price parameter assuming a lognormal distribution. see Train and Weeks 2005** [**https://doi.org/10.1007/1-4020-3684-1_1**](https://linkprotect.cudasvc.com/url?a=https%3a%2f%2fdoi.org%2f10.1007%2f1-4020-3684-1_1&c=E,1,QjclaLYEFVEqgTYhvFTdiXw9KkyyIc4lJJiRSSQe3uXitoTtzgRtXJV0rcMDupjabDweHkkT5iiGUHTEjjhxxLxXyjZstFOFBxX4OThllhRwxH1eYsGx&typo=1)

Our approach changed significantly, and we made changes throughout the text accordingly. We included Table 3 with the estimates in preference space with different model specifications (1: conditional logit; 2: mixed logit with out-of-pocket payment fixed and waiting times following a log-normally distribution and; 3: mixed logit with out-of-pocket payment and waiting times following a log-normally distribution). We also estimated a fourth model in WTP space with the same specifications of model 3 in preference space (Table 4). In addition, we included Table 5 where we contrast the WTP measures both in preference and WTP space.

**R2C10. Attach the DCE questionnaire as supplementary file**

We provide as supporting information the DCE survey in its original language (Hungarian) and a translation to English (S1_File).
