# Supplementary material for: Eliciting preferences for outpatient care experiences in Hungary: A discrete choice experiment with a national representative sample
Source: PLoS One. 2020 Jul 31;15(7):e0235165. doi: 10.1371/journal.pone.0235165 (PMC7394384; doi:10.1371/journal.pone.0235165)
Supplement: Attachment — Submitted filename: Response to Reviewers.docx [file pone.0235165.s005.docx]

**Response to Reviewers**

**Comments from Reviewer 1**

**R1C1. I have reviewed the responses to my comments and those of the second reviewer and am satisfied that the authors have made appropriate changes to the manuscript to address these. I look forward to the publication.**

Many thanks for your comments and time spent in reviewing our manuscript. We highly appreciate and value your contribution.

**Comments from Reviewer 2**

**R2C1. The methods sections still doesn't flow yet. When reporting DCEs, the first thing in the methods section should be attribute and levels selection or study setting followed by attributes and levels. Your paper starts with how data were collected before outlining what was being collected. There already exists a checklist for reporting Health related DCEs e.g. Bridges 2011
You can rearrange your methods section as follows
1. Attribute selection [or study setting can come before attributes and levels]
2. Attribute levels selection
3. DCE tasks and Experimental design
4. Preference elicitation
5. Instrument design [This is what you call survey in your paper. Write about the sections of your questionnaire]
6. Data collection [ This is where you include the data collection section including ethical approval]
7. Statistical analyses**

We rearranged the Methods section exactly as suggested by the Reviewer. We hope that the reading flows well now.

**R2C2. You aimed for sample size of 1000. What was this number based on? Rule of thumb, parametric method for calculating the sample size? State that in the text on how you derived the minimum sample size required.**

We clarified that the sample size of 1000 was based on rule of thumb. With a large sample size we could assign 250 people to each DCE block, and thus have sufficient confidence in model estimates.

**R2C3. The reason for excluding an opt-out or status quo is not convincing as in real market scenario a Hungarian patient can choose to opt-out of care or seeking care elsewhere. Though you assumed that the respondents would seek care, in your choice scenario, you did state that the health problem did not require immediate care. Patients should then have been allowed to opt-out as they can choose to delay care as it did not need immediate treatment. The opt-out could have possibly been labelled 'delay care' The repercussions for leaving out the opt-out is that it exacerbates hypothetical bias which further biases your WTP estimates. Therefore, you need to state this in your limitations. Furthermore, studies can still be designed in a way that includes and excludes the opt-out by for having two choice questions. For example, for those who choose the opt-out, you can ask them an additional question that forces them to choose between Alternative A or B only.**

In our choice scenario, we assumed that respondents would seek care to their concerning health problem at some point in time, regardless of hypothetically opting for delaying care before visiting a doctor. Although an opt-out option could have reduced bias in model parameter estimates, given that it mimics better a real market scenario, we believe that for the purpose of our study, which was understanding the preference weights of attributes of the care experience, this bias is acceptable in contrast with the implications of having included an opt-out option. For example, including an opt-out option could have increased the choice task complexity, and thus, increased the proportion of respondents that opt-out because of this reason, which is specially concerning to less educated respondents.

Also, findings of a study (DOI: [10.1371/journal.pone.0111805](https://dx.doi.org/10.1371%2Fjournal.pone.0111805)) with the objective of determining to what extent the inclusion of an opt-out option in a DCE may have an effect on choice behavior found small differences between the forced and unforced choice model. This increased our confidence in not including an opt-out in this study. Notwithstanding,  we are aware that studies can still be designed in a way that include and excludes the opt-out by for having two choice questions, as suggested by the Reviewer. We will consider this suggestion in future choice elicitation studies.

**R2C4. Line 198 "it would jeopardise a better understanding on respondents’preferences if a large number of respondents choose the opt-out option". This sentence would be better rephrased as "including the opt-out would not provide much information. However, this exposes the DCE to hypothetical bias as in real market scenario patients can opt-out of care or delay care.**

We followed the recommendation of the Reviewer and the revised the sentence as follows:

“We did not incorporate an opt-out or a status quo option, given that in the task instructions provided to respondents we assumed that they would have to seek care because of a concerning health problem at some point in time. Although an opt-out option could have reduced bias in parameter estimates, given that in real market scenario patients can opt-out of care or delay care, a forced choice method may lead to more thoughtful responses [1].”

**R2C5. Line 386. "Left bias". Did you mean "left-right" bias. In the model specification line 278, you state that where B0 is an alternative-specific constant that indicates respondents’ preference for consultation A vs. consultation B. But in your results tables 3,4,5 you define alternative specific constant as constant of choosing alternative B. This is a bit confusing. Is your alternative specific constant for Alternative A or Alternative B? Confirm**

We clarified this aspect in the text, following the comment of the Reviewer.

**R2C6. You have not stated in the statistical analyses section how you calculated your relative importance of attributes. You have provided information on how to calculated choice probabilities (preference weights) and WTP estimates but not relative importance estimates.**

Mistakenly, we used the terms ‘preference weights’ and ‘relative importance’ interchangeably, which is not correct. In our analysis, we have not computed relative importance estimates. Throughout the text we clarified this aspect, removing all references to ‘relative importance’. Thank you for raising this issue.

**R2C7. Tables 3,4,5, For the dummy coded variables, could you also state what the base levels were in the tables?**

Thank you for this comment. Given that the attributes that were dummy coded (A_3_ to A_6_) can be easily interpreted as having a positive experience in that attribute relative to not having a positive experience, we have decided to leave the Tables as they stand. However, we added base level information in the footnotes of the Tables.

**R2C8. Line 465 "The standard deviation of the WTP estimates were high, suggesting a large preference heterogeneity across respondents" did you mean large heterogeneity in WTP estimates? Rectifying the wording used**

We rectified the wording.

**R2C9. By using the term ‘independent random’ throughout the text, do you mean ‘random and normally distributed’?**

We did mean ‘random and normally distributed’. Where applicable, we clarified this in the text.

**R2C10. line 507 "To compute estimates in WTP space we used Stata’s user-written mixlogitwtp module". You had already mentioned this in the statistical analyses section**

This information repetition is part of the footnotes to Table 4, so that readers who have only the time to quickly scan the manuscript’s Tables, can have a better understanding of the statistical analysis used to derive those estimates.

**R2C11. Check your interpretation of WTP estimates, they have to be relative to the base. e.g Patients were willing to pay.**

Thank you for raising this issue. We have made changes throughout the text to bring clarity to the interpretation of the WTP estimates.

**R2C12. Your discussion section is very weak. Authors need to be a bit detailed in discussing their findings in light of their research objectives and compare their finding with other studies in similar settings. Also, the authors should have some strong policy recommendations. They have attempted these, but it is too weak. The discussion section needs to be strengthened**

We are sorry to know that following the previous rounds of review, the Reviewer still has this opinion in regard to the discussion of our findings. We believe that the discussion of our findings are aligned with the objectives of the study. However, we understand the critique of the Reviewer in regard to comparing our findings with those of other studies. To our knowledge, this is the first choice elicitation study focusing on aspects of the care experience using a set of standardized patient-reported experience measures to develop the choice tasks. We were not able to find a large number of elicitation studies to compare our results with; and those that we found were just too different and focusing on different measures (e.g. patient satisfaction). Partly because of sufficient evidence to contrast our findings with, we were cautious with the policy implications of our study. We do, however, pinpoint several policy implication to the Hungarian health care system, such as advocating for a wider involvement of citizens’ voice in setting the health agenda, having citizens’ and patients’ preferences partly steering health care organizations resources allocation, and supporting for systematic data collection of patients’ preferences and its use to inform the decisions of policy-makers encompassed in a broader health system performance monitoring and assessment framework.

In addition, we do believe that after our study, other could follow. Given that our choice sets were based on the statements of standardized PREMs, future cross-national comparisons are possible and desirable.
